# Dual-Step Chemical Treatment of Wafer-Scale Metal–Organic Chemical Vapor Deposition Grown Monolayer Molybdenum Disulfides

**DOI:** 10.1021/acsnano.5c08927

**Published:** 2025-09-25

**Authors:** Juhwan Lim, Anh Tuấn Hoàng, Zhaojun Li, Tran Thi Ngoc Van, Jung-In Lee, Kihyun Lee, Nicolas Gauriot, Kyle Frohna, Takashi Taniguchi, Kenji Watanabe, Bonggeun Shong, Kwanpyo Kim, Samuel D. Stranks, Jong-Hyun Ahn, Manish Chhowalla, Akshay Rao

**Affiliations:** † Cavendish Laboratory, 2152 University of Cambridge, Cambridge, CB3 OHE, U.K.; ‡ Department of Materials Science and Metallurgy, 2152 University of Cambridge, Cambridge, CB3 OFS, U.K.; § Department of Chemical Engineering and Biotechnology, 2152 University of Cambridge, Cambridge, CB3 OAS, U.K.; ⊥ School of Electrical and Electronic Engineering, 26721 Yonsei University, Seoul, 03722, Republic of Korea; ¶ Department of Materials Science and Engineering, 8097 Uppsala University, 75103 Uppsala, Sweden; ∥ Department of Chemical Engineering, 34954 Hongik University, Seoul, 04066, Republic of Korea; ◆ Department of Physics, 26721 Yonsei University, Seoul, 03722, Republic of Korea; ● Center for Nanomedicine, Institute for Basic Science (IBS), Seoul, 03722, Republic of Korea; ■ Research Center for Materials Nanoarchitectonics, 52747 National Institute for Materials Science, Tsukuba 305-0044, Japan; ▼ Research Center for Electronic and Optical Materials, 52747 National Institute for Materials Science, Tsukuba 305-0044, Japan

**Keywords:** metal−organic chemical vapor deposition, transition metal dichalcogenides, chemical treatment, defect passivation, photoluminescence enhancement

## Abstract

Two-dimensional (2D) transition metal dichalcogenides (TMDs) possess distinct optical and electronic properties, making them promising candidates for optoelectronic applications. Recently, major advances in the wafer-scale growth of TMDs using the metal–organic chemical vapor deposition (MOCVD) have enabled their integration with standard electronics. However, such materials continue to suffer from defects and unwanted doping, which lower semiconductor performance, as exemplified by poor photoluminescence (PL) yield. Chemical treatment protocols have been shown to improve the PL yield in exfoliated and CVD-grown materials. Here, using PL, Raman microscopy, X-ray photoemission spectroscopy (XPS) and density functional theory (DFT) calculations, we develop chemical treatment protocols for wafer-scale MOCVD-grown monolayer MoS_2_. The postgrowth treatment uses sulfide and TFSI-based ionic salts delivered via a solution process. We demonstrate a substantial PL enhancement ranging from 23 to 50 times, depending on the underlying MOCVD growth method of the MoS_2_. We present design rules for tuning chemical treatment protocols, depending on the defect densities and doping levels, allowing for successful passivation and large PL enhancements across different growth conditions. Our results demonstrate the versatility of these chemical treatment protocols and their potential to improve PL in device-relevant wafer-scale MOCVD-grown monolayer TMDs.

## Graphical abstract

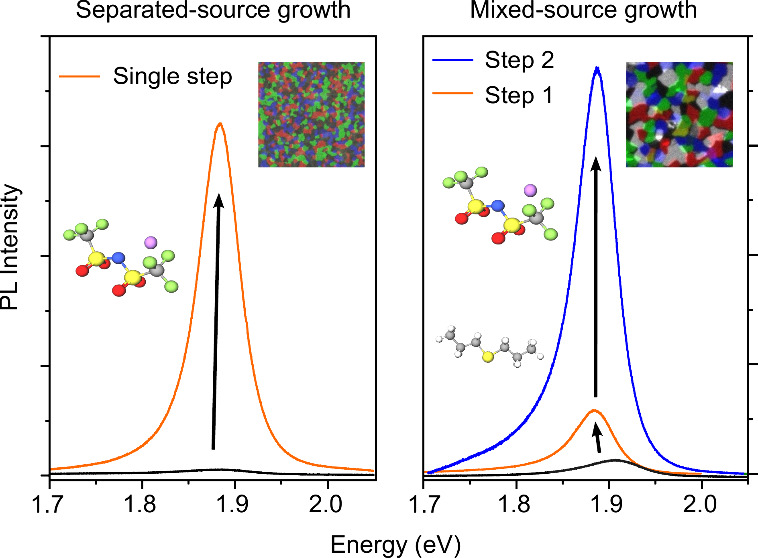

Monolayer TMDs with their direct bandgap, mechanical flexibility and versatility for chemical functionalization are promising material for ultrathin optoelectronic applications such as photodetectors, optical sensors, solar cells, and light-emitting devices.^1–3^ Recent advancement in wafer-scale growth shows the possibility of scalable optoelectronic applications for MoS_2_.^4–6^ Among various synthesis techniques, metal–organic chemical vapor deposition (MOCVD) is the most promising approach to obtain wafer-scale transition metal dichalcogenides (TMDs). MOCVD provides the advantage of a back-end-of-line (BEOL) compatible process, and many efforts are ongoing to improve the method, mainly for minimizing amorphous carbon contamination and reducing the reaction temperature.^7–10^ As new growth techniques evolve, the postprocessing of films grown by different methods also becomes a crucial issue for integrating MOCVD-TMDs into devices.

As an archetype on group-6 semiconducting TMD, MoS_2_ stands out as one of the most promising candidates for both ultrathin electronic and optoelectronic applications including photodetector and light-emitting diodes (LED).^11–14^ In addition, the photoluminescent property of MoS_2_ enabling *in situ* monitoring of chemical redox interactions or catalytic reactions on its surface, including hydrogen evolution reaction (HER).^15,16^ However, they typically show extremely low photoluminescence quantum yield (PLQY), reported as less than 1% in ambient conditions, due to the trion-based exciton quenching and various nonradiative recombination pathways induced by defects.^17–20^ Numerous efforts have been made to understand the nature of the radiative recombination process and identified effects from atomic defects, electron doping, excessive electrons on the surface, and substitute atoms on various TMDs.^19,21–26^ Monolayer TMDs have several different classes of defects.^27–30^ For example, MoS_2_ grown by chemical vapor deposition (CVD) exhibits numerous defects depending on the particular growth method, including single-atom vacancies originating from nonuniform vapor pressure and line defects such as grain boundaries due to the insufficient nucleation density and random crystal orientation during the growth process.^31–34^ Therefore, a systematic approach is required to build a strategy for enhancing the PL of MoS_2_ using novel growth methods.

Chemical treatment or electrostatic doping has been employed as one method to control the surface electrons or trion population to enhance the PL intensity of TMDs. The treatment involving *p*-dopants or negative-bias in field effect doping reduce the surface electron density and trion-mediated nonradiative processes.^18,35–43^ Among chemical approaches, H-TFSI (bis­(trifluoro­methane)­sulfonimide) and Li-TFSI (lithium bis­(trifluoro­methyl­sulfonyl)­imide) treatments on mechanically exfoliated TMDs show very large PL enhancements.^26,44–46^ Additionally, thiol-analogues have been introduced to address sulfur defects by filling sulfur vacancies.^47,48^ Both chemical approaches or combined methods passivate the defects and neutralize the surface, thereby increasing the population of neutral excitons and enhancing PL in MoS_2_ prepared by either mechanical exfoliation or CVD.^44,48,49^

Here, we report the first investigation (to the best of our knowledge) into chemical treatment on wafer-scale monolayer MoS_2_ films, with the effect of chemical passivation being strongly influenced by the underlying MOCVD growth methodology. By a thorough understanding of the characteristics of defects, doping, and action of the passivating chemicals, it is possible to design rational passivation routes that enhance the PL of wafer-scale MOCVD grown films by levels similar to PL enhancement achieved in mechanically exfoliated MoS_2_ flakes (23–50×).

## Results and Discussion

We studied wafer-scale (4-inch) MoS_2_ film grown by two different MOCVD methods – (1) using separated precursor source (SS) of transition metal and chalcogen, or (2) using mixed precursor source (MS), see Figure 1A. Conventionally, the SS method has been used for MOCVD growth of TMDs. However, amorphous carbon contamination is a major concern. Although chalcogen-hydride gases (i.e., H_2_S, H_2_Se) have been used to address these issues, requiring a high-temperature process (i.e., 1000 °C) makes them incompatible with back-end-of-line integration. Additionally, the hydrides are extremely toxic, thus causing the high cost for rigorous safety, which is another drawbacks.^50,51^ The MOCVD method utilizing the MS approach converts from individual precursors to ligands that contain both Mo and S elements, thus providing a new pathway to minimal amorphous carbon formation while keeping the advantage of a low-temperature process (i.e., 600 °C). The MoS_2_ film obtained by the MS approach shows improved uniformity and reduced defect density, which suppresses *n*-doping compared to the sample obtained by the SS method, as has been shown before.^6,52^ In both methods, molybdenum hexacarbonyl (MHC) and dimethyl sulfide (DMS) are used as precursors for molybdenum and sulfur, respectively. In contrast to H_2_S, DMS is less toxic and, therefore, garners the attention of the industry. In addition, the use of DMS allows minimization of the gas-phase parasitic reaction that can affect the uniformity and quality of TMDs. In the separated-source (SS) method, MHC is kept in its original solid form, creating an unstable flux. This results in an undesirable nonuniform nucleation distribution, leading to small grain size and variations in quality among different batches. In contrast, the mixed-source (MS) method provides a controllable way to introduce Mo flux by dissolving MHC in DMS, which creates the Mo­(CO_5_)­((CH_3_)_2_S) ligand.^52^ Therefore, this approach enables the modulation of nucleation density and leads to larger grain size. We measured the grain size of each growth method by dark-field TEM imaging using JEM-2100Plus (JEOL) system operated at 200 kV.^6^ The dark-field TEM images on the right side of Figure 1A clearly depict the different grain sizes of films from the two methods. SEM images taken during growth also demonstrate the larger grain sizes of the MS method (Figure S3). Importantly, the MS method promotes MoS_2_ growth dominantly on the substrate, eliminating the formation of amorphous carbon contamination, which is a primary concern in the MOCVD process (detailed explanation of the thermodynamics of the growth method is provided in Figure S4). The use of the MS method for the MOCVD process provides a better MoS_2_ quality (i.e., amorphous carbon-free, low sulfur vacancies, and reduces the number of grain boundaries), bringing MoS_2_ closer to practical application.^53^

**Figure 1:**
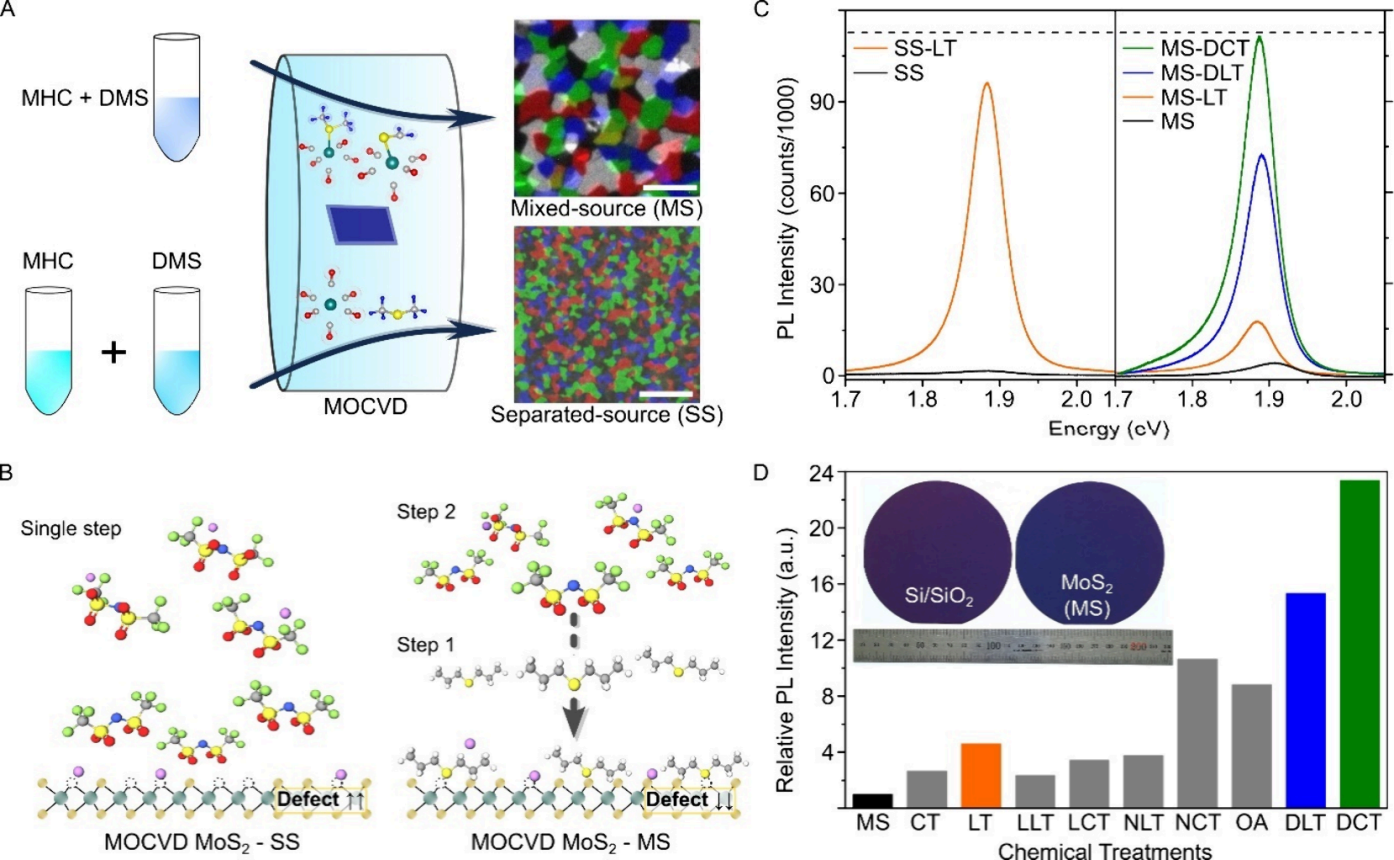
Chemical treatment for photoluminescence (PL) enhancement of MOCVD-grown MoS_2_. A. Schematic of two growth methods and dark-field transmission electron microscopy (TEM) images from each growth condition. Scale bar for the inset figures: 2 μm. B. Schematics of chemical treatment for MOCVD-grown monolayer MoS_2_, grown with two different method. MOCVD-MoS_2_ film named SS has grown with separated sources, and the film MS has grown with mixed-source. Single Li-TFSI treatment works for SS film (higher defect density) and dual chemical treatment process of dipropyl sulfide (DPS) followed by Li-TFSI works for MS film (lower defect density). C. Representative PL spectra for untreated (SS, MS), Li-TFSI treated (SS-LT, MS-LT), and dual-step chemical treated (DPS followed by Li-TFSI/Ca­(TFSI)_2_ denoted to MS-DLT, MS-DCT) MOCVD-MoS_2_ samples, measured at an excitation power of approximately 5 × 10^2^ W cm^–2^. The dashed line is an instrumental detection limit. D. General illustration of PL enhancements on MS film by different chemical treatments. PL intensity was normalized to the intensity of the pristine sample (MS). (CT: Ca­(TFSI)_2_, LT: Li-TFSI, LLT/LCT: Li_2_S followed by Li-TFSI/Ca­(TFSI)_2_, NLT/NCT: Na_2_S followed by Li-TFSI/Ca­(TFSI)_2_, OA: Oleic acid, DLT/DCT: dipropyl sulfide­(DPS) followed by Li-TFSI/Ca­(TFSI)_2_ treated). Inset images show 4 in. growth of MS-MoS_2_ films on Si/SiO_2_ wafers.

Figure 1B illustrates the generic scheme for the chemical treatment process on the MOCVD-MoS_2_ films in this study. All chemical treatment processes are conducted post growth, with the chemicals delivered to the films via the solution phase. As we develop below, the different growth methods require distinct chemical treatment approaches for enhancing PL. While the single Li-TFSI treatment enhances the PL of SS film, a dual-step sequential chemical treatment with dipropyl sulfide (DPS) followed by Li-TFSI enhances the PL of MS film to a similar level of Li-TFSI treated SS sample.

Figure 1C shows the PL spectra of each (SS, MS) when untreated and treated. To ensure reliable and repeatable measurements with a high signal-to-noise ratio, the excitation density and integration time were fixed for all measurements (see experimental methods for details). This allows for an accurate comparison of PL counts between different samples and treatment conditions. For the SS sample (left column), a single Li-TFSI treatment enhanced PL up to 49× (SS-LT), which is comparable to the previous reports using mechanically exfoliated samples.^44^ The MS sample (right column) having a lower defect density and larger grain size (details in Figure 1A) exhibits higher intrinsic PL intensity. However, the effect of a single Li-TFSI treatment on the MS sample (MS-LT) is around 3.5×, which is much lower than the effect for the SS sample. This was improved to 15× by pretreatment of DPS (MS-DLT) and further enhanced to 23× by dual-step treatment of DPS and Ca­(TFSI)_2_ (MS-DCT), giving a final PL intensity comparable to the intensity of Li-TFSI treated SS samples (MS-LT) (see experimental methods for details on treatment protocols). This highlights the intrinsic difference in the nature of the MoS_2_ film arising from different growth methods, which require distinct chemical treatment strategies for PL enhancement. The PL of the SS sample becomes brighter with a dual-step treatment of DPS followed by Ca­(TFSI)_2_ (Figure S2), while the effect of DPS pretreatment is more noticeable in the MS sample. When the domains coalesce, line defects can form, causing localized strain that impacts the band energy of the MoS_2_ film.^54,55^ As a result, variations in the defect density could lead to differences in the shifting direction of the PL peak position after chemical treatment.

Figure 1D shows the PL enhancement for MS-MoS_2_ films using various chemical treatment protocols. We studied the effect of inorganic compounds (lithium sulfide (Li_2_S), sodium sulfide (Na_2_S)) and dipropyl sulfide (DPS) as a prepassivation agent, and Li-TFSI and Ca­(TFSI)_2_ as *p*-doping agents. Additionally, we also tried oleic acid (OA) as a mild *p*-doping agent.^43^ (All chemicals are introduced in Figure S1.) The DPS treatment followed by Li-TFSI and Ca­(TFSI)_2_ shows the most significant PL enhancement for MS-MoS_2_. The inset figure depicts the 4 in. growth of MS-MoS_2_ film on Si/SiO_2_ wafer. The film exhibits uniformity, in both mechanical and optical characterization conducted through AFM, PL spectra, and Raman spectra measurements. Additionally, the transient absorption spectra indicate negligible defect-mediated low-energy transitions (Figures S5–S6).

To understand the difference in defects and doping between the SS and MS grown films, we turned to density functional theory (DFT). Figure 2 shows the energy bands and density of states (DOS) of three different types of MoS_2_: pristine, with lower sulfur vacancies (3% S_vac_, comparable to MS), and with higher S_vac_ (6% S_vac_, comparable to SS). The analysis of the partial density of states (PDOS) indicates that the pristine MoS_2_ has a band gap of approximately 1.81 eV, consistent with previous density functional theory (DFT) studies and experimental observations.^56–58^ In the presence of sulfur vacancies (S_vac_), midgap states are induced within the MoS_2_ bandgap. These new states act as local or transition states between the valence band maximum (VBM) and conduction band minimum (CBM). These new midgap states induced by sulfur vacancies cause a stronger *n*-doping effect at higher S_vac_ concentrations. This shows the intrinsic doping nature is different depending on different growth methods and implicate different chemical treatments on the surface would be necessary for enhancing the PL.

**Figure 2:**
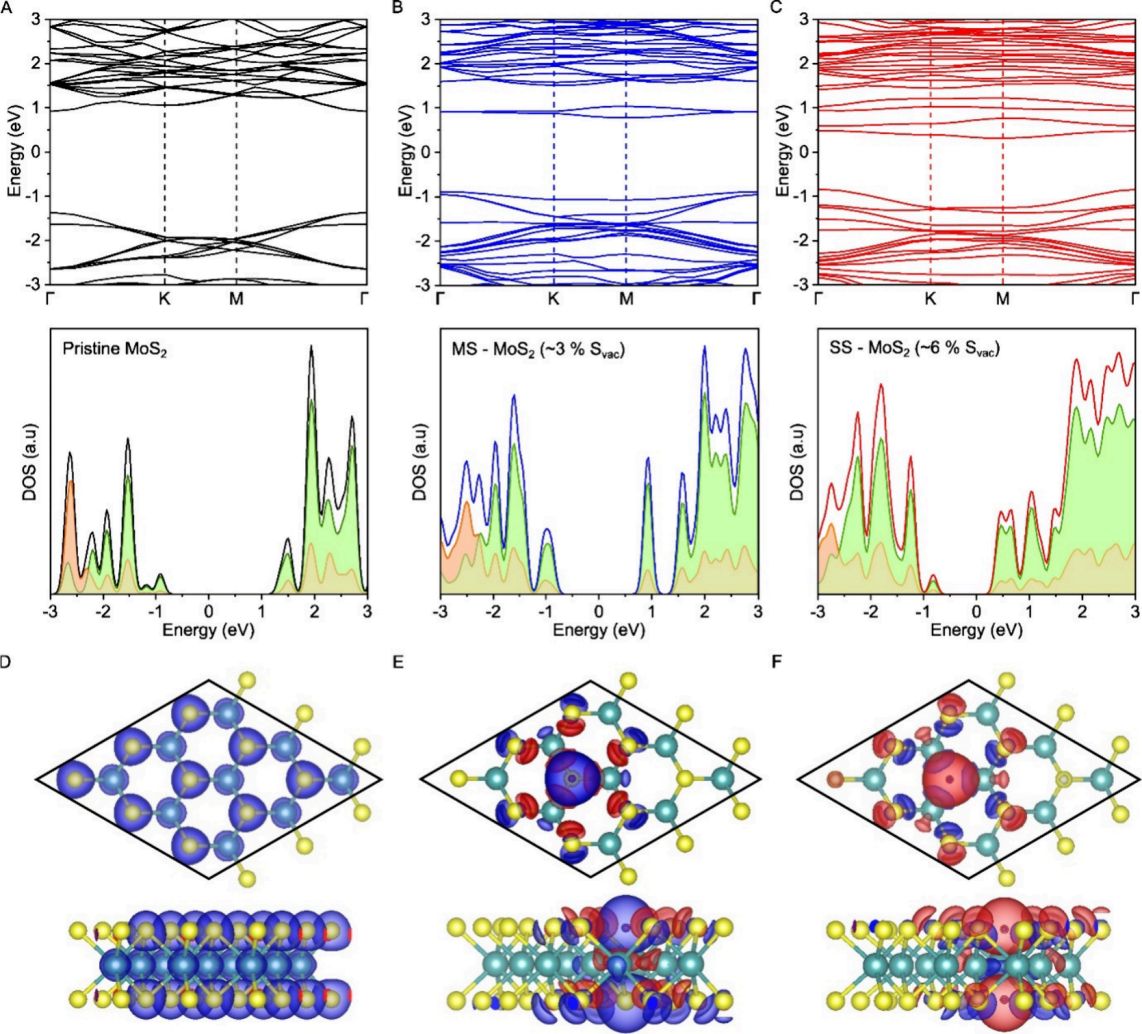
Band energy and DOS of pristine MoS_2_, and MoS_2_ with lower defect density and higher defect density. A-C. Band energy and density-of-state of pristine MoS_2_, low sulfur vacancies MoS_2_, and high sulfur vacancies MoS_2_, respectively. (At the lower column, green shaded: partial DOS of Mo atoms; orange shaded: partial DOS of S atoms; black, blue, and red lines represent the total DOS.) D-F. Partial charge density isosurfaces of pristine MoS_2_, low S_vac_ MoS_2_, and high S_vac_ MoS_2_ surfaces.

Figure 3A presents the PL spectra of untreated and Li-TFSI treated MoS_2_ prepared by three different methods: mechanical exfoliation (ME), MOCVD-SS, and MOCVD-MS. Before the treatment (lower panel), the MS sample exhibits the highest PL intensity with a blue-shifted peak position (1.9 eV) which is attributed to the emission of the neutral-exciton,^18,59^ while the ME and SS film show lower PL intensity and a PL peak at lower energy (around 1.87 eV) attributed to higher trion concentrations resulting from higher concentration of sulfur vacancy and grain boundaries. The Li-TFSI treatment enhanced the PL intensity of both ME and SS samples by approximately 50×, inducing a blue-shift in the PL peak by around 0.1 eV. This indicates that Li+ cation works as *p*-dopants, reducing the trion concentration and enhancing the PL, consistent with previous study^44^ (Table S1). However, for the MS sample, the PL enhancement is modest (3.5×), and the PL is red-shifted by around 0.11 eV. The PL intensity is uniformly distributed both before and after Li-TFSI treatment, and the intensity trend between ME, SS, and MS remains consistent with multiple measurements with different films or spots (Figure S7–8).

**Figure 3:**
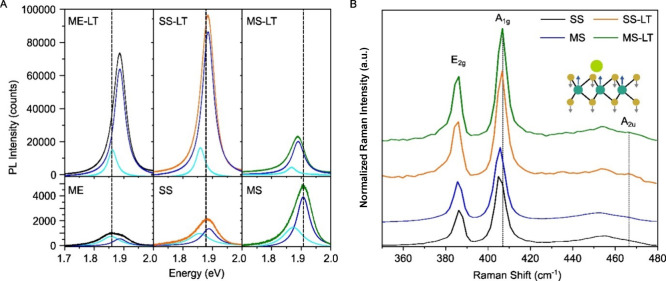
Li-TFSI treatment on MoS_2_. A. PL spectra of the untreated sample (ME for mechanically exfoliated, or SS, MS for as transferred sample, in bottom raw) and Li-TFSI treated (top raw) of each MoS_2_. All spectra deconvoluted by trion (cyon) and neutral exciton (blue) peaks. B. Raman spectra of Li-TFSI treated MoS_2_ in A. Inset image shows the Raman A_2u_ mode of MoS_2_.

Figure 3B presents the Raman spectra of untreated and Li-TFSI-treated MOCVD-MoS_2_ samples (SS, MS). The major Raman modes, including in-plane E_2g_^1^ mode at around 388 cm^–1^, and A_1g_ mode at around 405 cm^–1^ are observed for all untreated or treated samples (MS, SS).^60^ It is shown that the E_2g_ peaks of SS and MS samples are located at 405.59 and 404.83 cm^–1^, respectively, which presents both samples showing comparable strain properties in the constructed ε-n space within the Pos­(A_1g_) vs Pos­(E_2g_) plot of within the standard deviation of exfoliated MoS_2_ (Figure S9).^60^ With Li-TFSI treatment, the A_1g_ mode blue-shifts to 407 cm^–1^ with a new Raman A_2u_ mode at 468 cm^–1^ emerging for both SS and MS samples (Figure S10). The A_1g_ Raman mode is sensitive to electron doping, and the A_2u_ mode originates from nonsymmetry due to the presence of cations.^44^ Therefore, it is plausible that the interactions between the lithium cation and the MoS_2_ surface are similar in both samples, consistent with a previous study. Similar Raman spectra were measured at Ca­(TFSI)_2_ treatment (Figure S11). However, the other role of Li-TFSI emerges in the MS sample, a modest enhancement and a red-shift of PL peak, suggesting that although the general methodology of chemically driven PL enhancement has similar effects, the different nature of defects in films grown using different methods could lead to varied effects on the material, demanding a tailored study for different growth methods.

In Figure 4A, we investigated the effect of Li-TFSI on the MS-MoS_2_ surface by stacking a thin layer of mechanically exfoliated hexagonal boron nitride (h-BN) on top of the MoS_2_. The h-BN layer has a thickness of around 5–6 layers from the peak position of Raman spectra (1364.5 cm^–1^) (full Raman spectra presented in Figure S12).^61^ The MoS_2_ area covered by h-BN (blue spot in the inset in Figure 4A) exhibited comparable PL intensity and peak position but a narrower full width at half-maximum (FWHM) of the peak compared to the noncovered MoS_2_ (orange spot in the inset in Figure 4A). The thin layer of h-BN serves to protect the surface of MoS_2_ while allowing remote charge transfer doping through the h-BN flake, from the chemical dopants on the topmost surface to the MoS_2_ beneath.^62^ We observed enhanced and red-shifted PL when from the Li-TFSI treatment on a bare spot (orange spot/spectra), similar to Figure 3A. However, the red-shift was suppressed in the presence of h-BN on top, while PL was still enhanced (blue spot/spectra).

**Figure 4:**
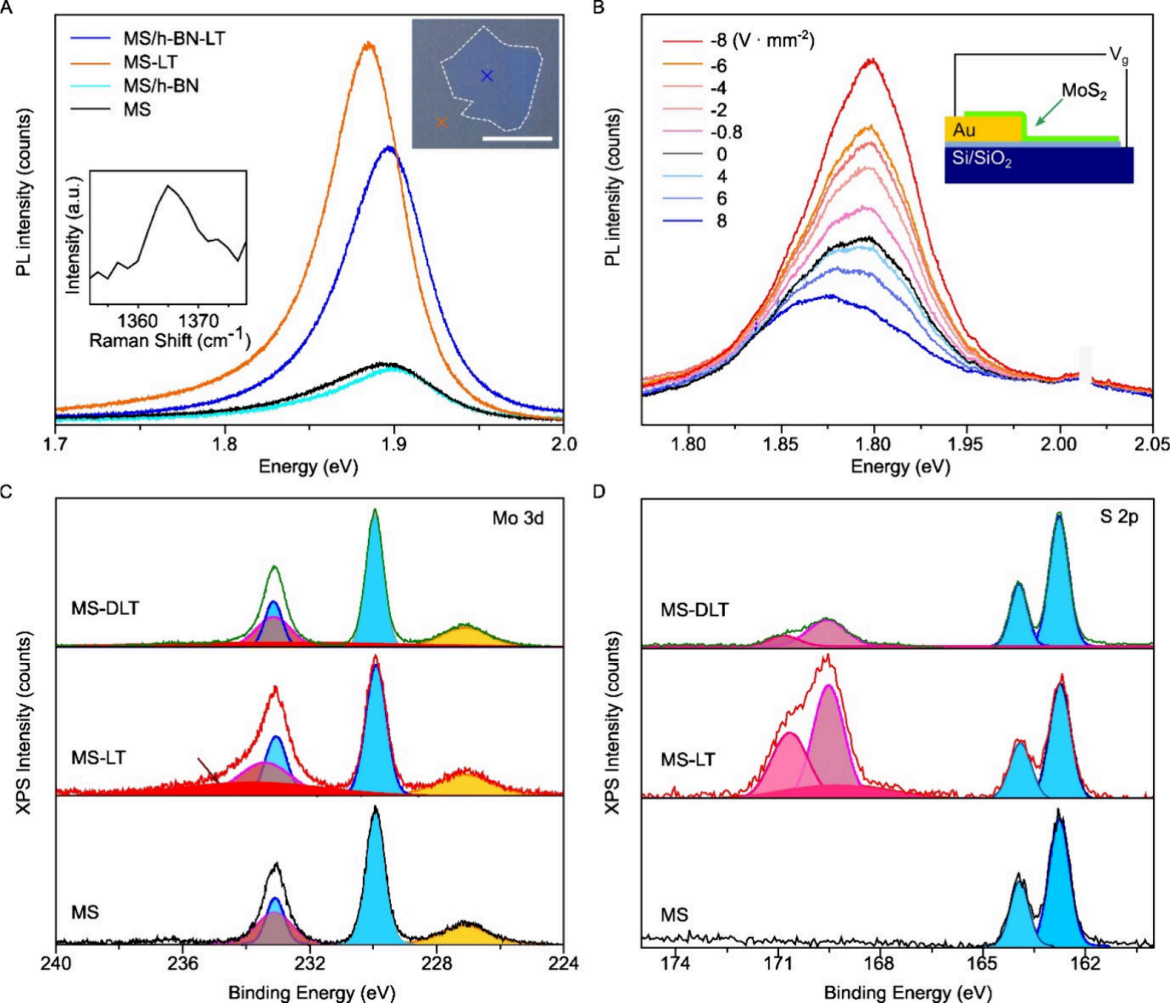
Li-TFSI treatment on MS-MoS_2_. A. PL spectra of h-BN covered the MS film. The inset graph shows the Raman spectra of h-BN. The inset image shows an optical image of the h-BN covered region, and the arrow shows the measurement point (blue: h-BN covered MS-MoS_2_, orange: uncovered MS-MoS_2_) B. Electrostatic PL modulation. Inset image shows the measurement schematic. C–D. XPS spectra of untreated (MS), Li-TFSI only treated MS-MoS_2_ (MS-LT), and DPS followed by Li-TFSI treated MS-MoS_2_ (MS-DLT) C. Mo 3d, and D. S 2p.

In Figure 4B, we employed electrostatic gating to modulate PL properties by controlling the background doping.^18,59^ We transferred MS-MoS_2_ on SiO_2_ (300 nm)/Si^++^ substrate with prepatterned Au electrode and measured PL with controlling the bias voltage (*V*_g_). The PL intensity decreased and red-shifted (from 1.9 to 1.88 eV) when *V*_GS_ > 0 V, while it increased and slightly blue-shifted when *V*_GS_ < 0 V. This result is consistent with previous reports,^18,59^ showing that the existence of a controllable background electron concentration on the MS sample can enhance the PL intensity by *p*-dopants, albeit accompanied by a very small blue-shift of PL peak position. Therefore, the red-shift observed in the PL spectra from the Li-TFSI treatment, in contrast to the consistent peak positions observed in the electrostatic gating or h-BN encapsulation experiments, suggests that the direct treatment of Li-TFSI on the surface may induce other effects on the MS-MoS_2_ surface that lead to the red-shifting of the PL peak. This aligns with oleic acid (OA) treatment, which is a simple long-chain acid known to be a weak acid to stabilize colloidal quantum dots by passivating dangling bonds. It has been reported while OA may not effectively protonate the monolayer TMD surface compared to Li-TFSI/Ca­(TFSI)_2_, it can reduce *n*-doping by passivating sulfur vacancies.^43,63^ In Figure S13, the treatment with OA exhibits a comparable enhancement in PL intensity to the Li-TFSI treatment while it did not result in a red-shift of the PL peak. Furthermore, we measured PL lifetime to compare the effect of DPS, Li-TFSI, and Ca­(TFSI)_2_ (Figure S14). The DPS treatment shows a shorter or very similar PL lifetime, while both Li-TFSI and Ca­(TFSI)_2_ treatments result in a longer lifetime, suggesting a trap-mediated exciton recombination process.^19,49^ This longer lifetime differs from the Li-TFSI treatment on exfoliate MoS_2_, which exhibited a shorter PL lifetime.^44^ DPS and thiol analogues including 2-furanmethanothiol (FSH) molecule passivate trap states.^44,46,49^ These results highlight the positive role of DPS in MS samples and suggest that the choice of chemicals should be carefully considered, not only for doping control but also for passivating various types of defects and trap states.^64^

In Figure 4C–D, we present X-ray photoelectron spectroscopy (XPS) analysis for Li-TFSI treated and DPS followed by Li-TFSI treated MS samples measured by ultrahigh-vacuum photoemission instrument (Escalab 250Xi). In the Mo 3d XPS spectra in Figure 4C, while three peaks from MoS_2_ (Mo 3d 3/2:232.9 eV, Mo 3d 5/2:229.8 eV, S 2s: 227.2 eV) appear, we observe another signal around 235.4 eV, which indicates the presence of oxidation with single Li-TFSI treatment.^7,65–67^ This oxidation is suppressed by prior DPS treatment. The XPS spectra of the S 2p in Figure 4D shows the signal from MoS_2_ (S 2p 1/2:164 eV, S 2p 3/2:162.8 eV) with the doublets of the TFSI anion at 169, 170.2 eV.^68,69^ Similar TFSI anion peaks are observed with Ca­(TFSI)_2_ treatment, and F 1s spectra show the presence of TFSI anion after LiTFSI treatment (Figure S15–16). The peak intensity for the TFSI residue on the surface is decreased at prior DPS treatment. The Li 1s signal in Figure S15 shows that lithium remains on MoS_2_ after both Li-TFSI and DPS followed by Li-TFSI treatment.^70^ However, it is challenging to confirm whether the physically adsorbed lithium forms a Li–S chemical bond or remains on the MoS_2_ surface and is oxidized during the measurement process. Our previous studies show that Li-TFSI cannot alter the electronic structure of WS_2_, and a divalent sulfur coordination is needed for effective defect passivation when the defects are neutral sulfur vacancies instead of charged defects.^46^ We propose that MS-MoS_2_ exhibits a reduced overall vacancy concentration with a lower proportion of charged sulfur vacancies from the favorable thermodynamics for the growth (Figure S4) and higher PL intensity with a blue-shifted energy (Figure 3A). As a result, the proportion of neutral defects would be higher at MS-MoS_2_. Accordingly, a sequential dual-passivation strategy employing dipropyl sulfide (DPS) followed by Li-TFSI treatment leads to more effective PL enhancement, consistent with our previous study.^46^ In contrast, SS-MoS_2_, which has a higher density of defects including both charged and neutral vacancies, responds well to Li-TFSI treatment alone, primarily due to its *p*-doping effect.^44^

Figure 5 presents the statistics of various chemical treatments on MS-MoS_2_ (original PL and Raman spectra of Li_2_S and Na_2_S treatment presented in Figure S17). We note that one to two points were measured per sample, and an untreated reference sample was measured alongside to ensure consistency. In the case of Li_2_S and Na_2_S, dual treatment is involved, both Li_2_S and Na_2_S have a strong tendency to undergo hydrolysis, forming strongly alkaline solutions. The significant red-shift observed is due to the formation of negative trion. The minimal effect of the Li_2_S followed by Li-TFSI treatment supports our hypothesis that divalent sulfur species are necessary for Li^+^ coordination. The overall trend of PL enhancement and peak shift remains consistent with Figure 1D, while the PL intensity in Figure 5A presents variation of up to 25% (Figure S20). We note that statistics of chemical treatments have generally not been well reported in the literature to date. Here, we present the statistics of each treatment to provide a guide for their reproducibility. A previous study has shown that TFSI-based salts with different cations (Cu^2+^, Mg^2+^, Ca^2+^, Na^+^, K^+^) exhibit similar PL enhancement, indicating a negligible correlation between cationic radii and PL tuning strength. However, we note, for instance, that when applying Ca­(TFSI)_2_, a divalent cation based ionic salt, the variation is higher than other treatments. This can be attributed to its lower ionic mobility compared to that of the Li^+^ ion. In Figure 5B, we compare the positions of the PL peaks. When we treated MoS_2_ with TFSI-based ionic salts, the red-shift became more prominent, while DPS treatment suppressed this red-shift. This variation may also be related to long-term stability, including air exposure during the measurement. Therefore, further studies investigating the stability of the treated films under conditions such as the treatment parameter, humidity, elevated temperature, and mechanical strain would be expected. Similar chemical passivation strategy is applicable to various TMDs, including MoSe_2_ and WS_2_. For WS_2_, both Li-TFSI and sequence-specific treatments enhance PL and mobility.^43,46,63,71^ Our findings regarding MOCVD-grown MoS_2_ suggest its broader applicability across TMDs with diverse growth methods.

**Figure 5:**
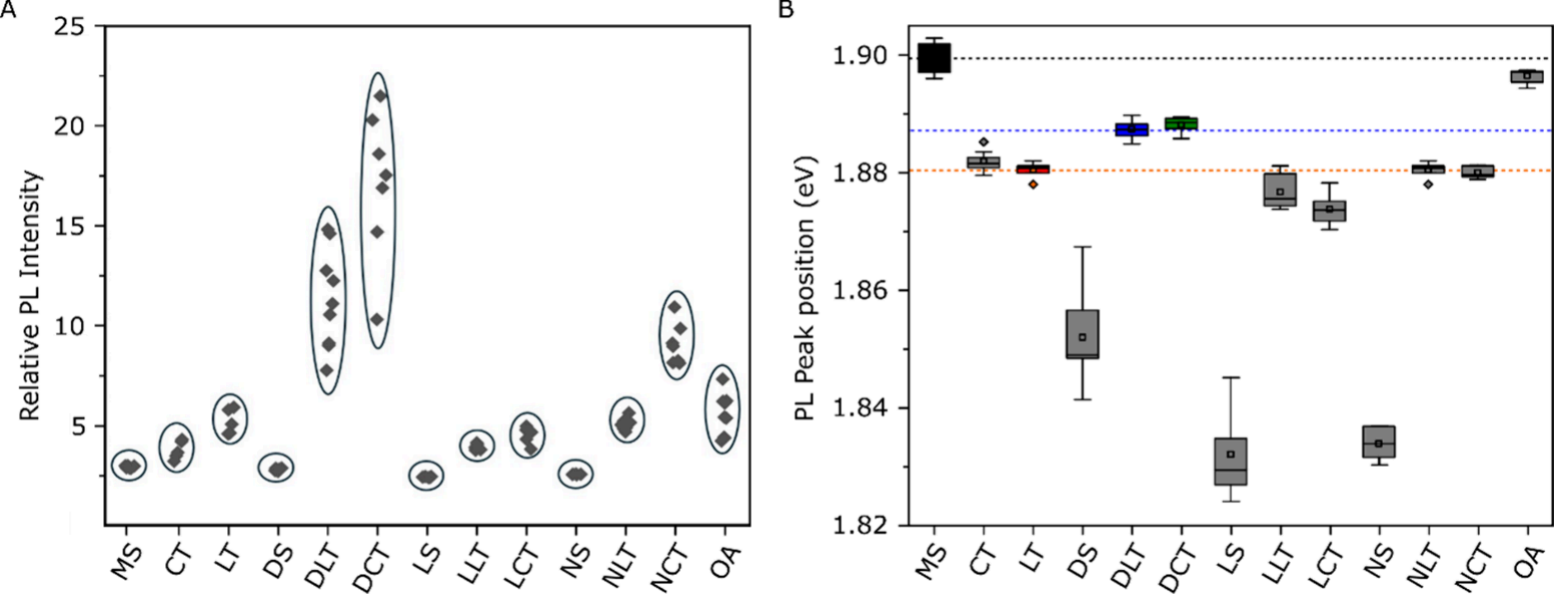
Statistics of various chemical treatments on MS-MoS_2_. A. PL intensity B. PL peak position.

## Conclusion

In summary, we investigated surface chemical treatment protocols to enhance the PL yields of wafer-scale MOCVD-grown monolayer MoS_2_. We showed that for MOCVD-MoS_2_ grown via the conventional separate source route, which results in higher defect densities and higher *n*-doping, TFSI-based ionic salt chemical treatment can greatly enhance the PL intensity. In contrast, MOCVD-MoS_2_ grown via the mixed source route, which shows lower defect densities with more neutral sulfur vacancies, does not respond as well to the TFSI-based chemical treatment. For these materials, we developed a dual-step chemical passivation strategy, providing an effective neutral defects passivation with the coordination between thiol and Li^+^. This led to a 23-fold improvement in PL yield from MOCVD-MoS_2_. Our results show that chemical treatment protocols to passivate defects and improve PL yield, which have so far been studied on exfoliated and CVD samples, can also be applied to wafer-scale MOCVD-grown materials. Furthermore, we presented design rules for how to tune chemical treatment procedures, depending on the defect densities and doping levels of the underlying materials, allowing for successful passivation and large PL enhancements across the board.

## Methods

### MOCVD Growth and Transfer

We used a home-built multiple heating zones MOCVD system to synthesize continuous monolayer MoS_2_ films. A 4 in. Si wafer with a 300 nm-thick SiO_2_ layer was cleaned with diluted hydrofluoric acid (HF: H_2_O = 1:20 v/v), deionized water, isopropyl alcohol, and dried with N_2_ gas before being placed into the MOCVD chamber. Molybdenum hexacarbonyl (MHC, MilliporeSigma 577766) and dimethyl sulfide anhydrous (DMS, MilliporeSigma 274380) were used as the Mo- and S-based precursors for the synthesis. For the separated-source method, 0.5 sccm MHC and 0.3 sccm DMS were injected independently into the chamber for 22 h. For the mixed-source method, 65 mg of MHC was dissolved entirely in 15 mL of DML. The mixture was then precisely injected into the chamber in two steps: starting with 1.0 sccm for 16 h, followed by an increase to 1.7 sccm for 6 h. A combination of 310 sccm Ar and 5 sccm H_2_ was used as a carrier gas for both methods to achieve a fully coalesced monolayer MoS_2_.

A thin layer of poly­(methyl methacrylate) (PMMA) was spin-coated on the top of the MOCVD-MoS_2_ film for the wet-transfer process. MoS_2_ films on Si/SiO_2_ were cut into appropriate sizes and floated on deionized (DI) water. Within an hour, the Si/SiO_2_ substrates detached from the MoS_2_/PMMA and submerged, while the MoS_2_/PMMA remained floating on the DI. This separation occurs due to the difference in hydrophilicity between the SiO_2_/MoS_2_ interface and the existence of the ionic sacrificial layer. Next, using the “scooping up” method, we transferred the floating MoS_2_/PMMA film onto the glass substrate. The samples were then carefully dried on a hot plate overnight and placed under vacuum for several hours to enhance adhesion. Finally, the PMMA layer was removed by washing the samples with sufficient amounts of acetone and isopropanol (IPA).

### Sample Preparation

#### Mechanical Exfoliation

We mechanically exfoliated MoS_2_ and h-BN using a PDMS-assisted method. First, we roughly exfoliated MoS_2_ crystal (2D semiconductor) and h-BN crystal using blue tape (Ultron systems) and then transferred them to the PDMS film. Flakes with suitable size, thickness (monolayer MoS_2_ and very thin-layered h-BN) and shape were identified and transferred onto designated substrates (thin glass substrate (170 μm thickness, Thorlab) or Si/SiO_2_ substrate with 300 nm SiO_2_. The monolayer MoS_2_ was confirmed by measuring the PL spectra.

#### Transfer of MOCVD-MoS_2_ (MS, SS)

The transfer is done by DI mediated wet-transfer.

### Chemical Treatment

We made and treated each chemical for chemical treatment studies following the procedures. All treatments were conducted in a nitrogen glovebox to ensure an inert environment. In the case of the dual-step treatment, we did the first chemical treatment, fully dried the sample, and then completed the second chemical treatment.

#### Li-TFSI and Ca­(TFSI)_2_

The solid form of each Li-TFSI and Ca­(TFSI)_2_ salt was bought from Sigma-Aldrich. Each salt dissolved in Methanol (Sigma-Aldrich) at a concentration of 5 mg/mL. The chemical treatment of MoS_2_ has been done by immersing the whole sample for 30 min, followed by drying, mild washing using Methanol (for removing excessive chemicals), and the sample was fully dried. All treatments were conducted in a nitrogen glovebox to ensure an inert environment.

#### Dipropyl Sulfide (DPS)

A 0.1 M solution of dipropyl sulfide was prepared by dissolving dipropyl sulfide powder in 1,2-dichloroethane solvent (Both from Signal Aldrich). We note that 1,2-dichloroethane (DCE) is a nonpolar medium known for its effectiveness in dispersing MoS_2_ nanosheets without inducing additional sulfur vacancies.^72^ Previous reports have shown that DCE may lead to mild *n*-type doping, as Cl atoms incorporated into sulfur vacancies, which may explain the slight red-shift observed in PL (Figure 1C).^73^ However, our data indicate that DPS treatment results in defect passivation rather than defect formation.

MoS_2_ sample was immersed in the prepared solution for 1 day. Then, sample went through mild washing using 1,2-dichloroethane, and dried. All processes were conducted inside the glovebox.

#### Na_2_S, Li_2_S

We made 0.1 M Na_2_S and Li_2_S solution by dissolving each salt (Signal Aldrich) in a methanol solvent. Treatment was done by immersing the sample for 1 day followed by gentle washing using methanol and drying in the nitrogen glovebox.

#### OA

Oleic acid (OA) has been used following the experimental procedure in a previous study.^43^

### Optical Characterization

The steady-state PL and Raman measurements were performed under ambient conditions by using a Renishaw inVia Raman microscope. The excitation laser source with a wavelength of 532 nm was utilized for both PL and Raman measurements. Prior to conducting any measurements, the spectrometer was calibrated using a silicon reference sample to correct for instrument responses and having a stable initial count to the silicon wafer.

Each treated sample was stored in a nitrogen-filled glovebox for less than 2 weeks and subsequently measured under ambient conditions. Each sample was exposed to air for less than 6 h during measurement. The PL enhancement remained consistent throughout this period, indicating that short-term exposure to air does not significantly compromise the treatment’s efficacy. This suggests that the treatment maintains reasonable stability under typical laboratory conditions.

During the measurements, the laser power was set to 0.05% (<0.5 μW) and focused onto the specific point of the flake using a 20× long working distance objective (NA = 0.40). The FWHM of the excitation beam is 678.3 nm, calculated using the NA (0.40) and excitation wavelength (532 nm). From the dark-field TEM image, we can postulate that we are collecting data from 4 to 5 grains of MS sample, and 9–10 grains of SS samples. The emitted light was collected in streamline mode and dispersed by a 1800 l/mm grating.

The time-resolved PL lifetime was measured by using a custom-built optical microscope setup. Excitation was provided by filtering white light using a 532 ± 10 nm band-pass filter. PL lifetimes were measured using TCSPC electronics after filtering out using a 660 ± 10 nm band-pass filter.

### Quantum Chemical Calculations

DFT calculations for gas-phase reactions were carried out using the Gaussian 16 suite of programs.^74^ The geometries of molecular precursors were optimized by using the B97D3 functional with a def2-tzvp basis set, except for Mo for which LANL2DZ pseudopotential was assigned. For the calculation of the Gibbs free energy values, a temperature of 700 °C and a pressure of 10 Torr were assumed.

DFT calculations for the MoS_2_ slab models were performed using Vienna ab initio simulation (VASP)^75^ package version 5.4.4. The monolayer of MoS_2_ with vacuum space of 15Å was used for calculations. For structural relaxation, the PBE functional^76^ with D3BJ dispersion correction^77^ was applied using projector-augmented wave methods. A cutoff energy of 350 eV and a 2 × 2 × 1 Monkhorst–Pack k-point mesh were used for relaxation and self-consistent calculations. For the PBE-optimized geometries, the projected density of state (PDOS) and band gap were obtained using HSE06 hybrid functional^78^ and a 9 × 9 × 1 Monkhorst–Pack k-point mesh.

## Supplementary Material



## Notes

S1: Structure of chemicals used in this study. S2: PL spectrum of dual-step chemical treated SS sample. S3: SEM images of MoS2 domains. S4: Thermodynamics for MOCVD growth. S5: Wafer-scale uniformity of MOCVD MoS2. S6: Optical properties of transferred MoS2 film. S7: Comparison of PL intensity between the exfoliated and MOCVD grown films. S8: PL mapping. S9: Raman fitting. S10–S14: Raman and PL of samples with and without chemical treatment. S15–S16: High resolution XPS scan. S17: PL of samples treated by Li2S and Na2S. S18–S20: Statistic of Raman/PL of samples treated with different chemicals (PDF)
