# Supplementary material for: Dual-Step Chemical Treatment of Wafer-Scale Metal–Organic Chemical Vapor Deposition Grown Monolayer Molybdenum Disulfides
Source: ACS Nano. 2025 Sep 25;19(39):34698–707. doi: 10.1021/acsnano.5c08927 (PMC12509315; doi:10.1021/acsnano.5c08927)
Supplement: Supplementary file 1 [file nn5c08927_si_001.pdf]

Supplementary information for

Dual-step chemical treatment of wafer-scale metal-organic chemical vapor deposition grown monolayer molybdenum disulfides

Juhwan Lim[1,2], Anh Tuấn Hoàng[4], Zhaojun Li[1,5], Tran Thi Ngoc Van[6], Jung-In Lee[2], Kihyun Lee[7,8], Nicolas Gauriot[1], Kyle Frohna[1], Takashi Taniguchi[9], Kenji Watanabe[10], Bonggeun Shong[6], Kwanpyo Kim[7, 8], Samuel D. Stranks[1,3], Jong-Hyun Ahn[4]*, Manish Chhowalla[2]*, Akshay Rao[1]*

[1] Cavendish Laboratory, University of Cambridge, Cambridge, CB3 OHE, UK

[2]Department of Materials Science and Metallurgy, University of Cambridge, Cambridge, CB3 OFS, UK

[3]Department of Chemical Engineering and Biotechnology, University of Cambridge, Cambridge, CB3 OAS, UK

[4]School of Electrical and Electronic Engineering, Yonsei University, Seoul, 03722, Republic of Korea

[5]Department of Materials Science and Engineering, Uppsala University, 75103 Uppsala, Sweden

[6]Department of Chemical Engineering, Hongik University, Seoul, 04066, Republic of Korea

[7]Department of Physics, Yonsei University, Seoul, 03722, Republic of Korea

[8]Center for Nanomedicine, Institute for Basic Science (IBS), Seoul, 03722, Republic of Korea

[9]Research Center for Materials Nanoarchitectonics, National Institute for Materials Science, Tsukuba 305-0044, Japan

[10]Research Center for Electronic and Optical Materials, National Institute for Materials Science, Tsukuba 305-0044, Japan

Juhwan Lim and Anh Tuấn Hoàng equally contributed to this work

*E-mail: ahnj@yonsei.ac.kr, mc209@cam.ac.uk, ar525@cam.ac.uk

Supplementary 1. Structure of chemicals.

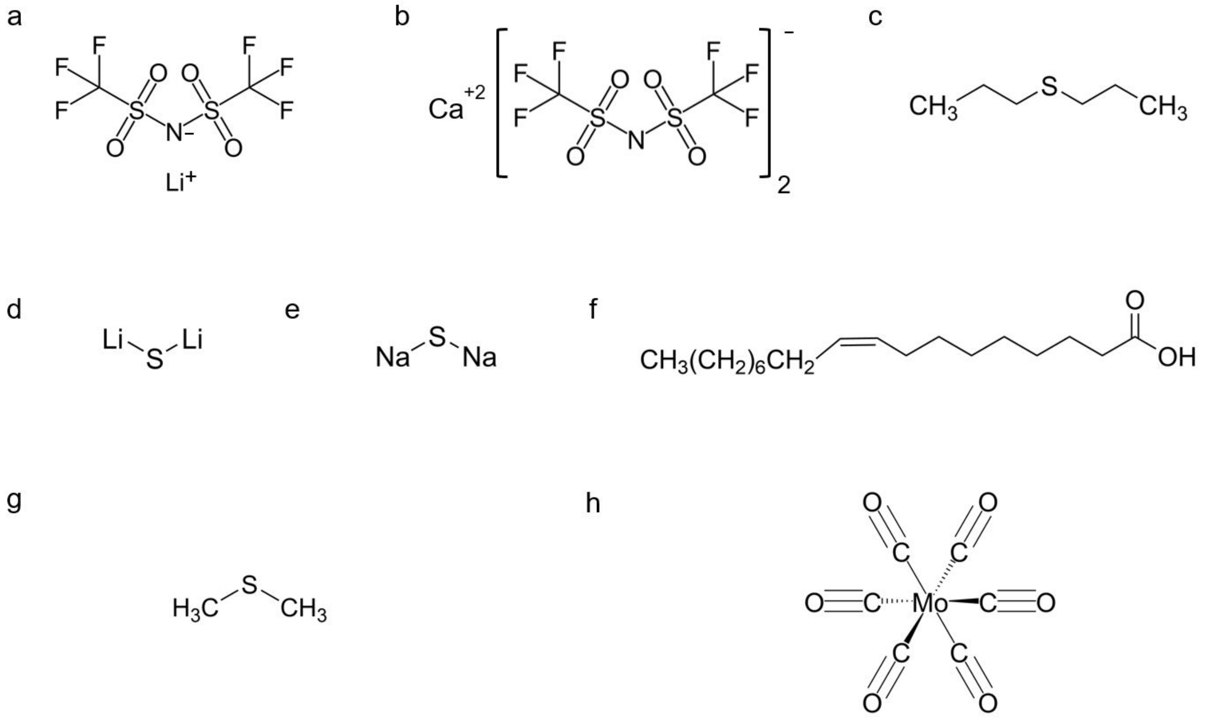

Fig. S1. Structure of chemicals in this study. a. Li-TFSI. **b.** $Ca(TFSI)_2$. **c.** Dipropyl sulfide (DPS). **d.** Lithium sulfide (Li_2S). **e.** Sodium sulfide (Na_2S). **f.** Oleic acid (OA). **g.** dimethyl sulfide anhydrous (DMS). **h.** Molybdenum hexacarbonyl (MHC).

Supplementary 2. Dual-step chemical treated SS sample

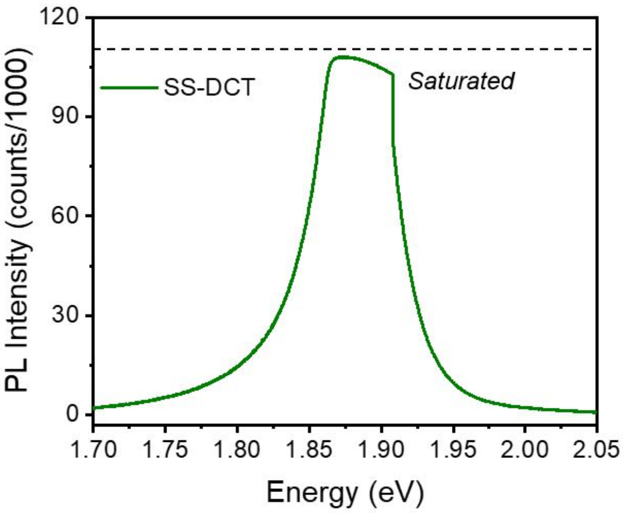

Fig. S2. PL spectra of dual-step chemical treated SS sample (DPS followed by $Ca(TFSI)_2$)

Supplementary 3. SEM image during the growth.

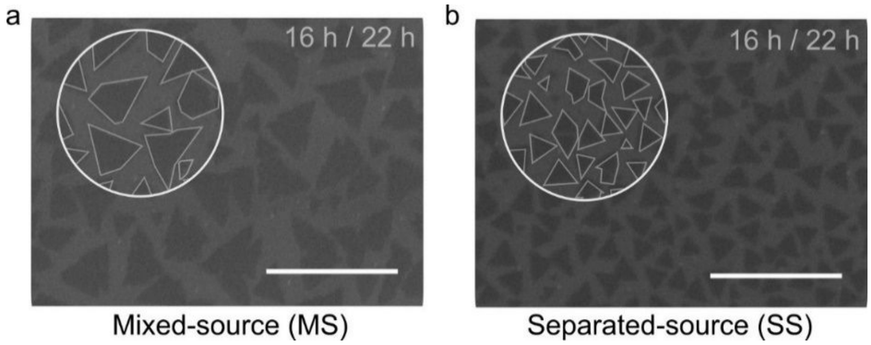

Fig. S3. Scanning electron microscopic (SEM) images of MoS_2 domains taken during the synthesis process. **a.** A sample from MS method and **b.** A sample from SS method. Sample taken out during the growth (at the first 16 h during 22 h long process) to visualize the discrete domains before coalescing to form continuous films. Scale bar = 2 μm

SEM images were taken during the process which we stopped the growth process at 16 h (instead of obtaining fully coalesced film after 22 h). Because this is during the growth, each grain is not merged yet. So, this figure shows the different nucleation density of each method and how each sample shows different concentrations of grains.

Supplementary 4. Thermodynamics for wafer scale MoS_2 growth.

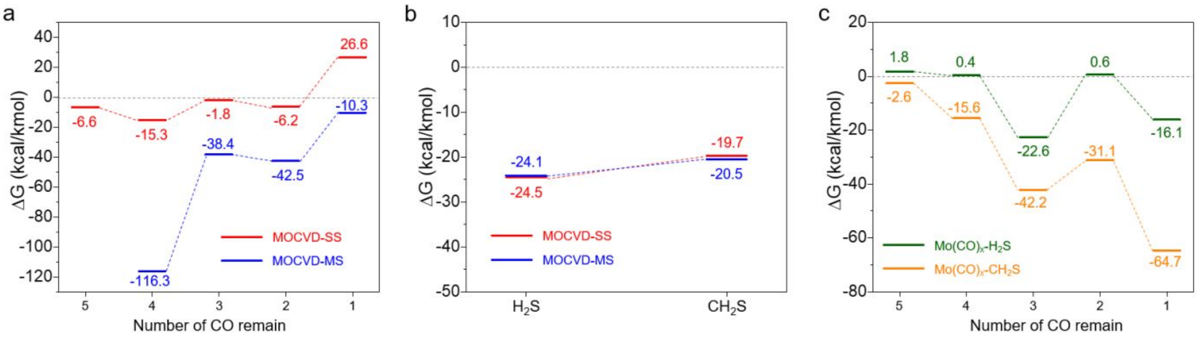

Fig. S4. a. Thermal decomposition of MHC using SS (red line) and MS (blue line) methods. **b.** Thermal decomposition of DMS using SS and MS methods. **c.** Reaction between decomposed products for MoS_2 formation.

Fig. S4 unveils a discovery of the MS method, which proves to be a significantly more effective approach. It aids in the complete and efficient decomposition of MHC, as opposed to the SS method, where MHC decomposition simply loses CO gradually. Fig. S4 a shows that the MS method helps to decompose MHC more easily and completely while MHC in the SS method is incompletely decomposed (MHC decomposition is simply lose CO gradually).

Reaction1. $Mo(CO)_6 \rightarrow Mo(CO)_{6-x} + xCO$ *(SS method)*

Reaction2. $Mo(CO)_5\text{-}DMS \rightarrow Mo(CO)_{5-y}\text{-}DMS + yCO$ *(MS method)*

Fig. S4 b showing DMS is completely decomposed with both method (due to high temp regime), and mainly becomes H_2S or CH_2S. Fig. S4 c shows the reactivity between Mo(CO)x with H_2S or with CH_2S. It seems the MoS_2 formation is mainly due to $Mo(CO)_x$ and CH_2S. It can explain why MoS_2 can be synthesized at “low temp” using DMS while high temp is required if using pure H_2S gas. Also, the reaction is more easy to happen with $Mo(CO)_1$; however, we can only get $Mo(CO)_1$ with MS method which is indicated in Fig. S4 a. Therefore it implies 1-precursor method can help to reduce defects, especially sulfur vacancies. This explains why high-quality MoS_2 can be synthesized at a low-temperature regime using DMS compared to the synthesis using pure H_2S as the sulfur precursor. The DFT data in Fig. 2 further supports that the sulfur vacancies can be reduced using the MS method.

Supplementary 5. 4-inch wafer scale grown MOCVD(MS)-MoS_2.

The optical image (adapted from Figure 1 D in the main manuscript) and AFM image shows the uniformly grown Monolayer MoS_2 thin film on SiO_2/Si wafer.

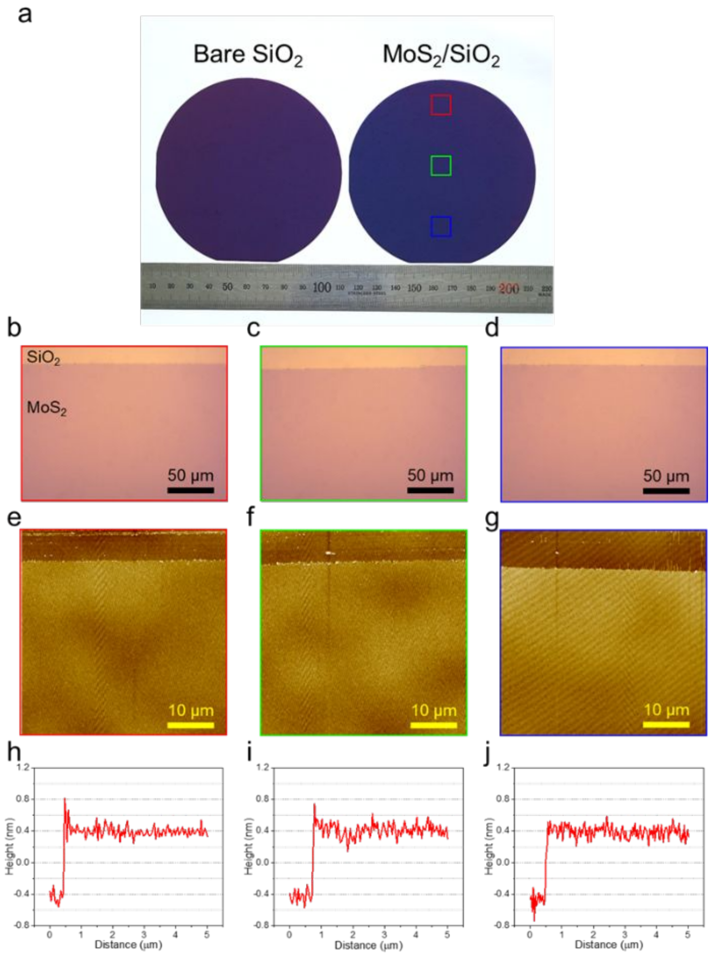

Fig. S5. MOCVD(MS)-MoS_2. a. Photographs of 4-inch wafer before (left) and after (right) growth. **b-g.** Optical microscopic images and corresponding atomic force microscopic (AFM) images of top, center, and bottom wafers, respectively(marked as red, green, blue square). **h-j.** Height profiles extracted from AFM images, showing the uniformity of monolayer MoS_2.

Supplementary 6. Optical property of transferred MOCVD-MoS_2 (MS) film on glass

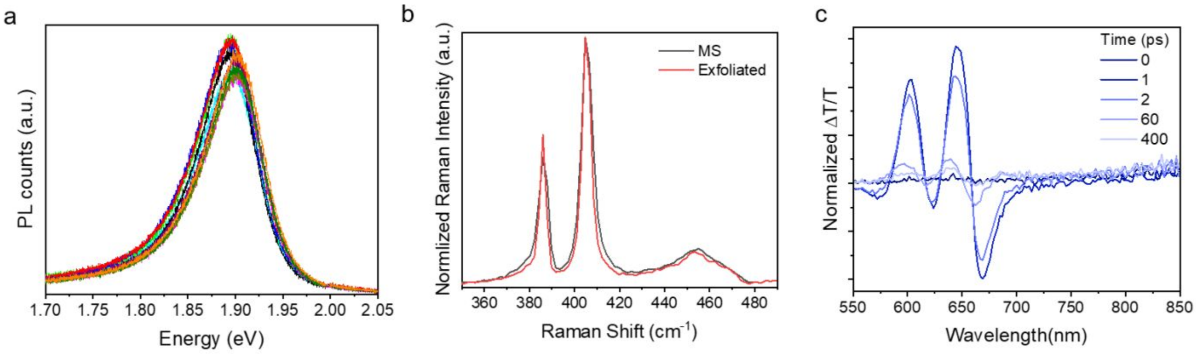

Fig. S6. Optical property of as-transferred MS-MoS_2 film. a. PL spectra from different spots **b.** Raman spectra of MS MoS_2 film (black) and mechanically exfoliated MoS_2 (red). **c.** Transient absorption graph of MS MoS_2 film

In Fig. S6 a, the PL spectra measured in MS-MoS_2 film exhibit peak positions ranging from 1.895 eV to 1.904 eV. Additionally, the Raman signals in Fig. S6 b display identical Raman peak positions compared to the mechanically exfoliated monolayer MoS_2. Fig. S6 c illustrates the transient absorption spectra, which shows no in-gap state at lower energy. It has been shown previously that in the presence of sulfur vacancies, a new spectral features arises at 730 nm, below the optical gap of the system[1,2]. This result indicates this material has relatively low defect density.

Based on this, we conclude that the transferred MS-MoS_2 samples have a comparable defect density and doping level to exfoliated flakes, making them suitable for our chemical passivation study.

Supplementary 7 – Comparison of PL intensity and Raman spectra of as-prepared (exfoliated or transferred) and Li-TFSI treated mechanically exfoliated (ME), and MOCVD-MoS_2 (SS, MS) films.

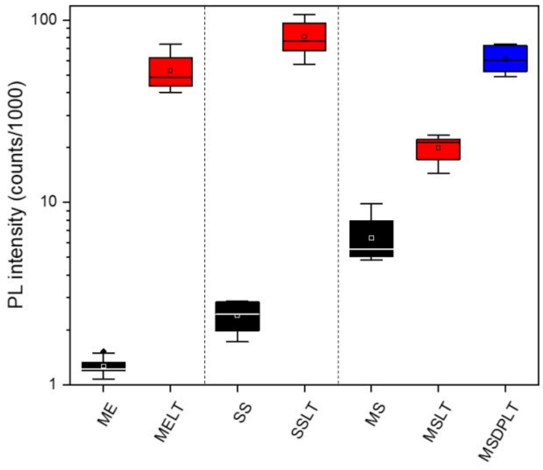

Fig. S7. Comparison of PL intensity of as-prepared (exfoliated or transferred) and Li-TFSI treated mechanically exfoliated (ME), and MOCVD-MoS_2 (SS, MS) film.

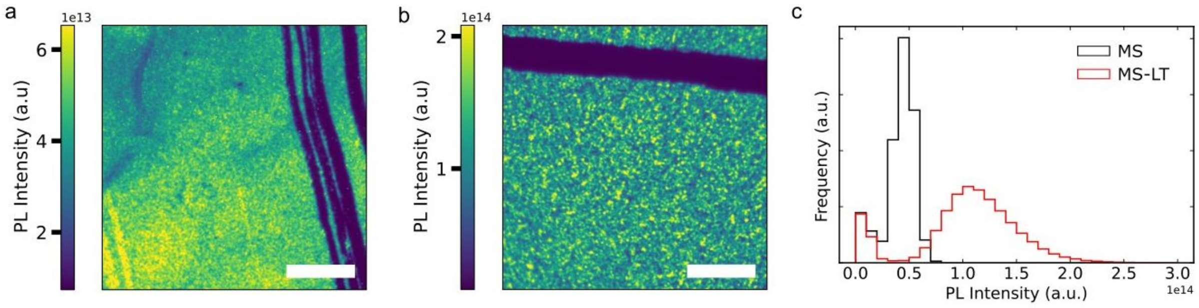

Fig. S8. PL mapping of MS-MoS_2. a. as-prepared film. **b.** Li-TFSI treated film (scale bar = 15 μm) **c.** Histogram of PL counts.

Sample	As prepared		Li-TFSI treated		Enhancement of Neutral Exciton Ratio (%)
	A_{Tri} (relative ratio, %)	A_{EX} (%)	A_{Tri} (%)	A_{EX} (%)	
ME	94.79 (64.0 %)	53.31 (36.0 %)	964.16 (17.5 %)	4545.83 (82.5 %)	46.5 %
SS	156.69 (55.1 %)	127.69 (44.9 %)	1046.34 (13.9 %)	6472.13 (86.1 %)	41.2 %
MS	218.24 (38.5 %)	349.33 (61.5 %)	251.04 (14.1 %)	1531.16 (85.9 %)	24.3%

Table. S1. Comparison of the ratio of neutral exciton (A_{EX}) and trion (A_{Tri}) before and after the Li-TFSI treatment (original spectra in Fig. 3A)

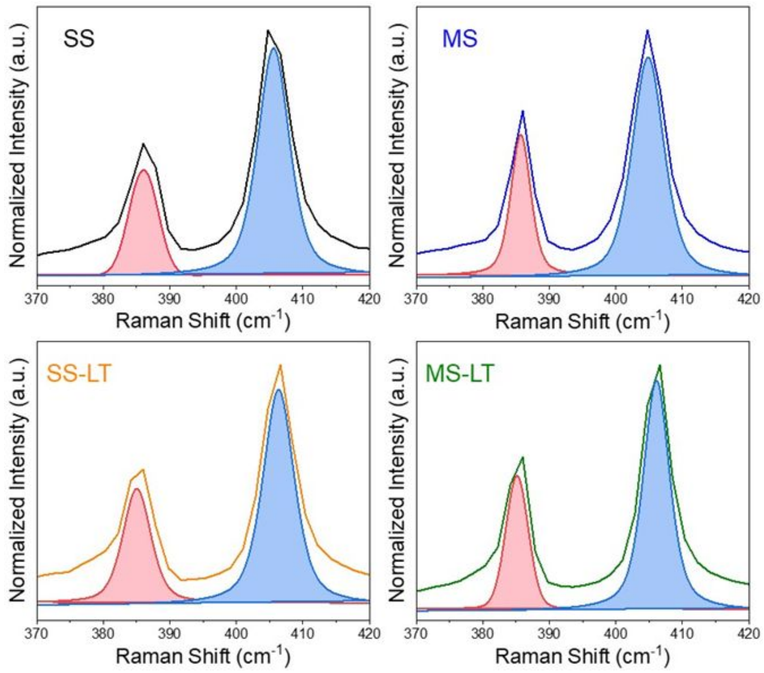

Figure S9. Raman fitting of SS, MS, SS-LT, MS-LT samples using the Voigt function.

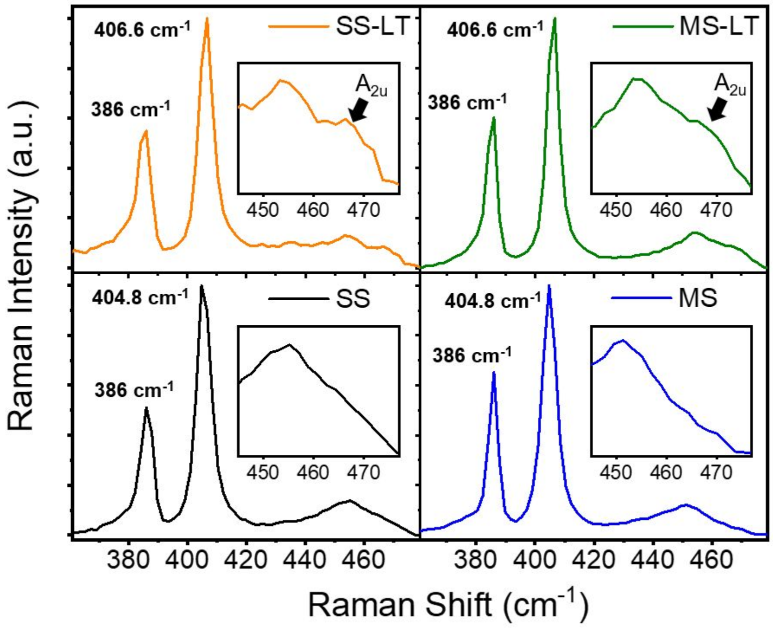

Fig. S10. Raman spectra of Li-TFSI treated, and non-treated MS/SS sample (in Fig. 3A)

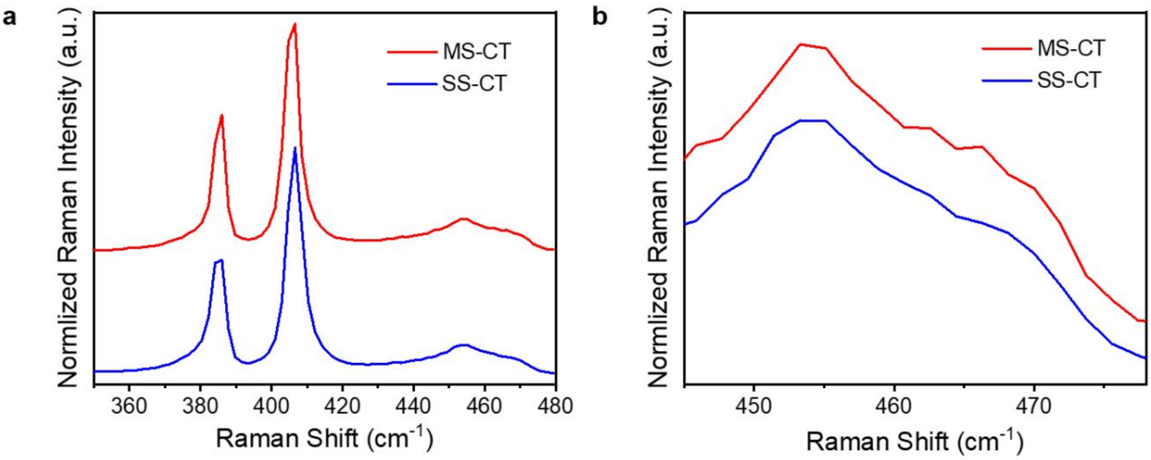

Fig. S11. Raman spectra of $Ca(TFSI)_2$ treated sample. a. Raman shift range of 350 - 480 cm^{-1} **b.** Raman shift range of 445 - 478 cm^{-1} (Magnified graph from **a**)

Supplementary 8 – Raman spectra of chemical treated MS-MoS_2 with h-BN on top.

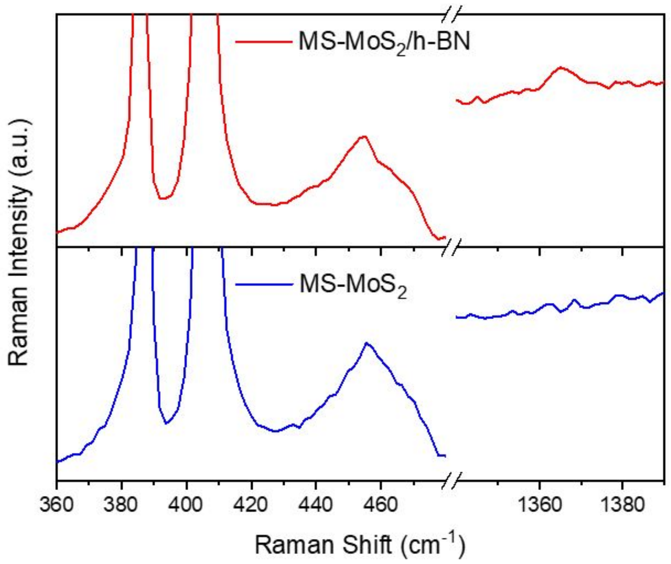

Fig. S12. Raman spectra of MS-MoS_2 and MS-MoS_2 covered thin h-BN layer on top after Li-TFSi treatment

Supplementary 9 – PL, Raman spectra of Oleic-acid treated MS-MoS_2 film

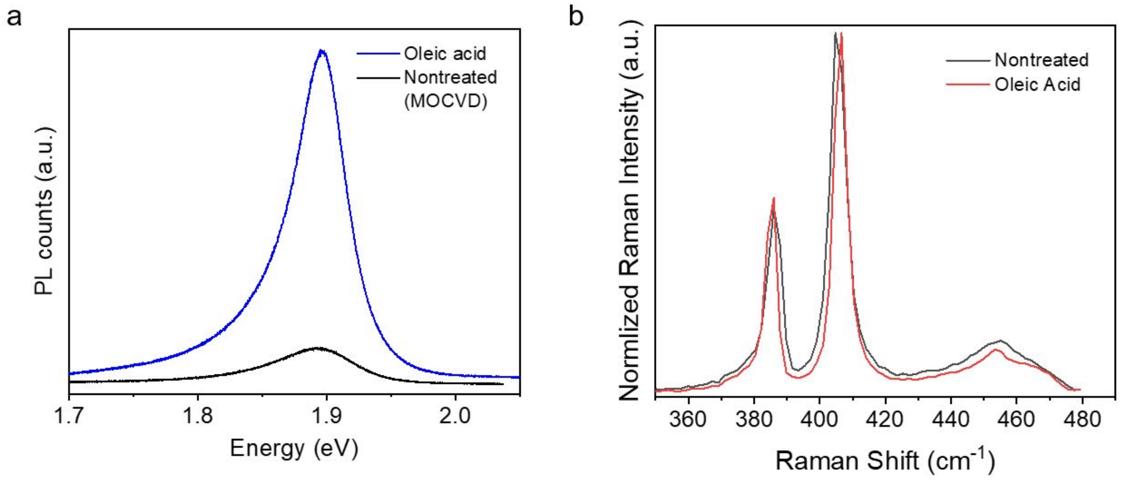

Fig. S13. PL, Raman spectra of Oleic-acid treated MS-MoS_2 film.

Supplementary 10 – PL lifetime measurement of DPS, Li-TFSI, and $Ca(TFSI)_2$ treated MS-MoS_2 film

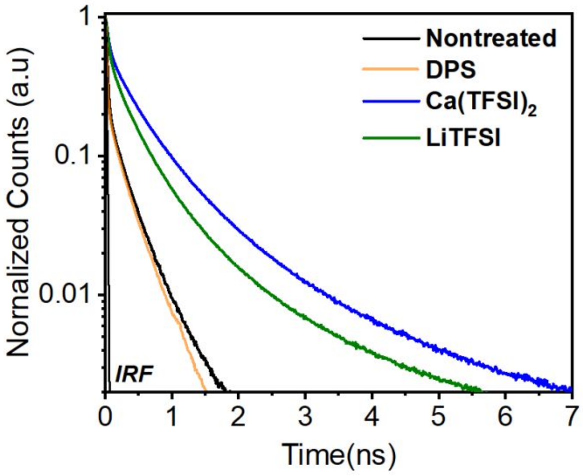

Fig. S14. PL lifetime of DPS, Li-TFSI, and $Ca(TFSI)_2$ treated MS-MoS_2

Supplementary 11 – XPS spectra of chemical treated film

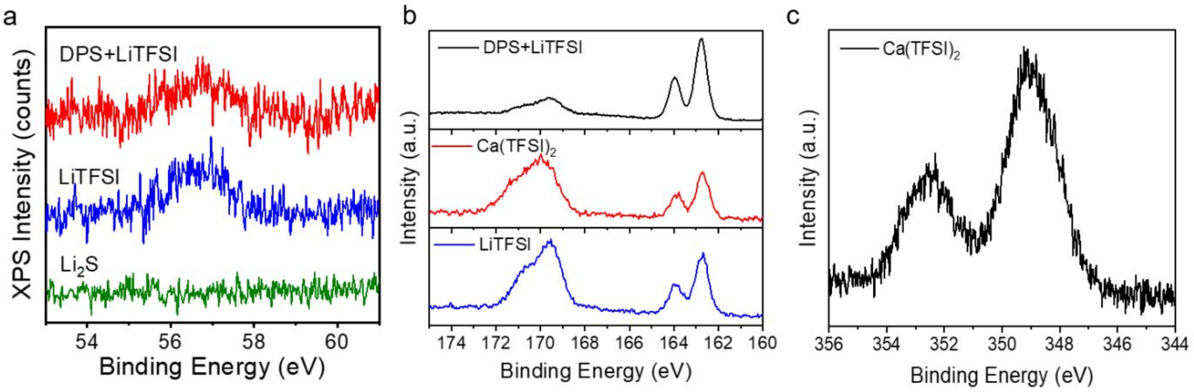

Fig. S15. XPS spectra. a. Li 1s after Li_2S, Li-TFSI, and DPS+Li-TFSI treatment **b.** S 2p at DPS+Li-TFSI treatment, $Ca(TFSI)_2$ treatment and Li-TFSI treatment. **c.** Ca 2p for $Ca(TFSI)_2$ treated MS-MoS_2

Fig. S15 b-c presents the signal from both sulfur and calcium from $Ca(TFSI)_2$ treatment shows the similar to Li-TFSI. The S 2p spectra shows the doublets of the TFSI anion signal at $Ca(TFSI)_2$ treatment, similar to single LiTFSI treatment. The Ca 2p signal shows the existence of calcium on the surface after the treatment, similar to lithium ion (blue line in a). Both peaks in Ca 2p can be assigned to CO_x compounds.

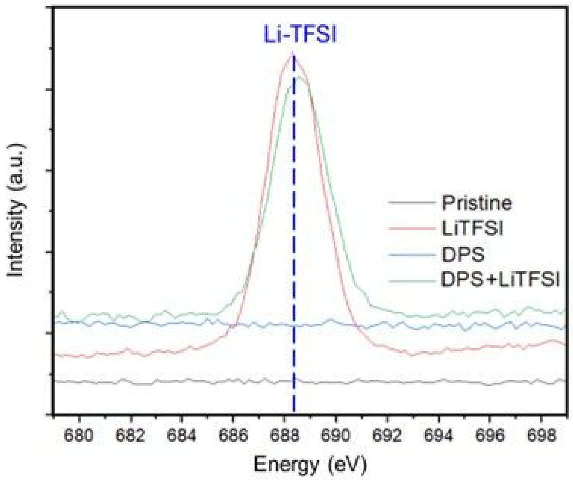

Fig. S16. XPS F 1s spectra.

Supplementary 12 – PL and Raman spectra for Li_2S, Na_2S, and dual-step treatment with Li-TFSI or $Ca(TFSI)_2$.

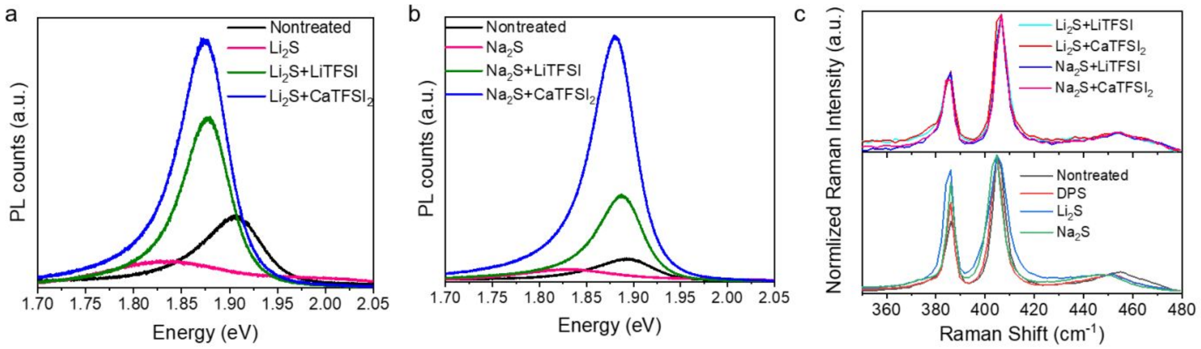

Fig. S17. Chemical treatment using Li_2S and Na_2S. a. PL for MS-MoS_2 with single Li_2S and Li_2S+LiTFS/$Ca(TFSI)_2$ dual chemical treatment. **b.** PL for MS-MoS_2 with single Na_2S and Na_2S+LiTFS/$Ca(TFSI)_2$ dual chemical treatment. **c.** Raman spectra for Li_2S, Na_2S, and dual-step treatment with Li-TFSI or $Ca(TFSI)_2$

Supplementary 13 – Raman analysis (E_{2g}-A_{1g} peak position differences) of MS-MoS_2 with various chemical treatments

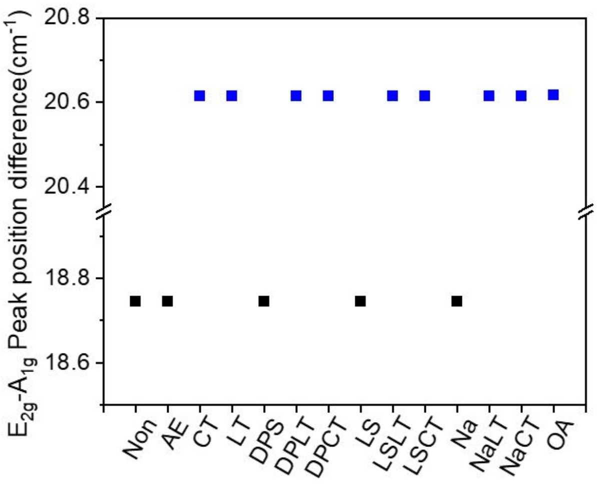

Fig. S18. E_{2g}-A_{1g} peak position differences between various chemical treatment

Supplementary 14 – PL counts of exfoliated MoS_2

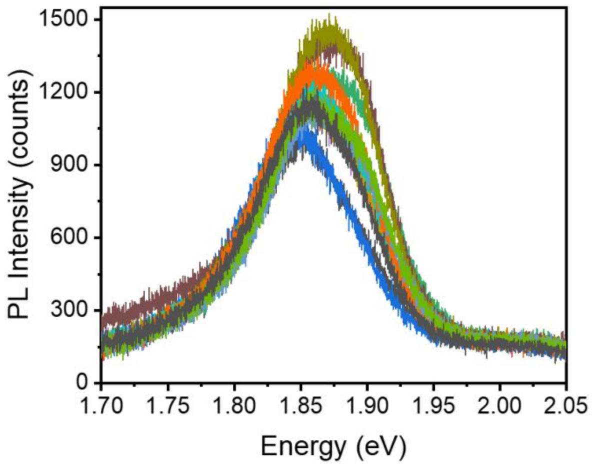

Fig. S19. PL spectra of mechanically exfoliated sample

Supplementary 15 – Box chart plot and number of data points for Fig 5A

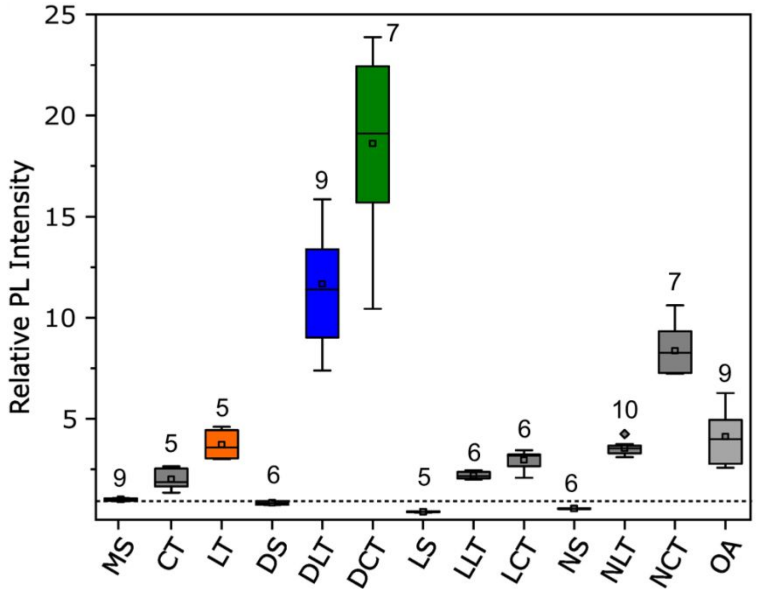

Figure S20. Box chart plot for Fig 5A and number of data points

References

1. Goodman, A. J., Willard, A. P. & Tisdale, W. A. *Phys. Rev. B* **2017**, 96, 1–6.

2. Bretscher, H. et al. *ACS Nano* **2021**, 15, 8780–8789.
